# Iodine concentration in tap water, mineral water, and coffee

**DOI:** 10.29219/fnr.v67.9517

**Published:** 2023-05-08

**Authors:** Monica Hauger Carlsen, Ellen Kielland, Maria Wik Markhus, Lisbeth Dahl

**Affiliations:** 1 Department of Nutrition, University of Oslo, Oslo, Norway; 2 Division of Chemical Food Safety, Norwegian Food Safety Authority, Oslo, Norway; 3 Department of Seafood and Nutrition, Institute of Marine Research (IMR), Bergen, Norway

**Keywords:** iodine, drinking water, mineral water, coffee, nutrient, food composition

## Abstract

**Background:**

Sufficient iodine intake is important for thyroid function and, particularly, among women of reproductive age. Water is a universal component of the diet and could be an important source of iodine. Iodine concentration in drinking water varies geographically. It is therefore of nutritional interest to explore the variation and the contribution of iodine from water and beverages.

**Objective:**

To analyze the iodine concentrations in tap water, mineral waters, and coffee from different regions of Norway.

**Design:**

Samples of tap water were obtained from different regions of Norway. Six brands of mineral water and several samples of coffee brews were sampled. The iodine concentration was determined by Inductively Coupled Plasma-Mass Spectrometry (ICP-MS).

**Results:**

Iodine concentration in tap water varied from below Limit of Quantification to 0.8 μg/100 mL. Five out of six brands of mineral water had low concentrations of iodine, and one brand had a concentration of 38 μg/100 mL. Iodine concentrations in black coffee brews were similar to the tap water. Adding milk or plant-based milk alternatives increased the iodine concentration.

**Discussion:**

Overall, iodine concentrations in tap water were generally low; however, variations were observed both for inland and coastal regions. A trend was seen for higher iodine concentrations in coastal region compared with inland region. For the average habitual iodine intake in Norway, tap water may not contribute significantly. One brand of mineral water could have considerable impact on iodine intake. Coffee does not contribute substantially more to iodine intake than tap water, unless the brew is added with milk or plant-based milk alternatives that contain iodine.

**Conclusion:**

This study adds new information about iodine dietary sources in Norway. While tap water and black coffee have limited impact due to generally low concentrations, one mineral water brand may contribute significantly to iodine intake.

## Popular scientific summary

- Drinking water is a universal vehicle for dietary minerals including iodine, however concentrations vary geographically.
- Water samples from Norway indicate generally low, but varying levels of iodine for both coastal and inland regions.
- One mineral water brand has a high concentration of iodine and may contribute significantly to intake.
- Iodine content in coffee is dependent on tap water concentrations.

Our diet is the source of multiple nutrients and non-nutrients. Nutrient concentrations may vary several folds within and between foods, due to natural, biological, or geographical variations. Iodine is one such micronutrient, distributed unevenly among food sources. In the Norwegian diet, iodine is found natural in high concentrations in lean saltwater fish and in medium concentrations in dairy products, due to the fortification of feed. Thus, in Norway, the most important iodine dietary sources are lean fish and dairy products, and other dietary sources contribute much less to the iodine intake (1–4).

In recent years, multiple dietary surveys have reported the risk of insufficient intakes of iodine in sub-groups of the population in Norway, especially in young girls and women of childbearing age (3, 5–7). Drinking water is a vehicle for nutrients, in particular minerals, including iodine. The concentration of iodine in tap water in Norway has been investigated, to our knowledge, in reports published by Hetland (8) and Simensen (9) in 1973, including water samples from 12 locations, and in 2004 by Dahl et al., including samples of drinking water from 15 locations (2).

Tap water and other beverages may contribute to the iodine intake. Beverages such as coffee and mineral waters are popular commodities in Norway. In the last national dietary survey, the average intake of coffee was 591 mL per day and 454 mL per day in men and women, respectively (10). The average intake of water was 1.1 L per day, of which the majority was tap water (10). Earlier estimations of iodine intake from water and beverages used food composition data partly borrowed from the Swedish Food composition database (1). As the iodine concentration of tap water varies with geographical locations (2, 8, 9), more data on possible natural variations are warranted.

The aim of the present study was to measure the concentration of iodine in tap water, mineral waters, and coffee samples from different geographical regions in Norway. This knowledge is important for the iodine food composition database and for estimating iodine intake from water and beverages in dietary surveys.

## Methods

### Sampling of tap water

The sampling of tap water was organized as a convenience sample during winter and spring 2019. Colleagues, acquaintances, friends, and family of the researchers in the study, living in geographically different regions of Norway, were invited by email to sample tap water from their home or workplace. Those who agreed to take samples were provided with a 10 mL sampling tube and written instructions. The water samples were to be collected between noon and midnight, and after the water had been left running for approximately 60 s. The collectors were also requested to provide information about date, time, and postal code/address of the sampling location. The samples were returned to the University of Oslo by prepaid postal envelopes and stored at room temperature until shipment to the laboratory at the Institute of Marine Research, Bergen.

### Sampling of mineral water

Five bottles of mineral waters (0.5 L) from different brands were purchased in May 2019 in a supermarket in Bergen, Norway. In addition, four more bottles of mineral water from one brand (Farris) were purchased in March 2020. The bottles were stored unopened at room temperature until analysis.

### Sampling and preparation of coffee

Several different brands of coffee were included based on marked share reports from AC Nielsen Norge (2018). Five different brands of brewed filter coffee, one type of instant coffee, three different types of capsule coffee, and two types of espresso coffees were purchased in supermarkets in Bergen and Oslo, during November 2018 and May 2019. Sampling of brewed filtered, instant, and capsule coffees was conducted in Oslo and Bergen.

To prepare one sample of filtered coffee, 60 g of coffee and 1 L of tap water were funneled using a filter coffee machine (Moccamaster). Three different batches of each of the five brands of filter coffee were prepared, resulting in 15 samples of filtered coffee in each round (November 2018 and May 2019). Approximately 50 mL coffee was transferred into a plastic tube and kept in a refrigerator (4°C) until analysis. Samples from Oslo were kept 1–2 days in refrigerator before shipment to Bergen. In Bergen, the coffee samples were kept in refrigerator for 2–4 days before analyses.

Instant coffee samples were prepared by mixing 4 g of instant coffee and 150 mL of boiled water in a cup. A total of three samples of instant coffee were prepared in each round (November 2018 and May 2019). Capsule coffee was prepared using a coffee machine (Nespresso) and the medium water volume as described for each capsule coffee type. The machine can make three different portion sizes of each coffee type, and the medium size was used, which corresponds to approximately 60 g of prepared coffee in each cup of the three types of capsule coffee prepared (Fortissio Lungo, Kazar, and Ristretto).

Approximately 50 mL of the prepared coffee was transferred into a plastic tube and kept in a refrigerator (4°C) for few days until analysis.

The espresso coffee samples were prepared using an espresso machine (Electrolux) in Bergen in May 2019. Nine grams of dry coffee and 30 g of tap water were used for the preparation of each sample. In total, six samples with two different brands were prepared and then transferred into a plastic tube and kept in a refrigerator (4°C) for few days until analysis.

In addition, a composite sample of the grounded beans of each filtered coffee brand, instant coffee, and espresso coffee was prepared by mixing the three sampled batches of coffee (dry matter). Equal amounts of dry coffee from the same brand were homogenized and pulverized before the sample was prepared for analysis. A total of eight composite samples were prepared.

A convenience sample of nine brewed caffè-lattes-added plant-based or lactose-free milk from three different coffee bars in Bergen was purchased in March 2020.

### Analysis of iodine content

Iodine was determined in samples of tap water, mineral water, and coffee by Inductively Coupled Plasma-Mass Spectrometry (ICP-MS) at the Institute of Marine Research (IMR). The laboratory is accredited according to NS-EN ISO/IEC 17025 (2017). Each water and prepared coffee sample was added 100 μL of tetra methyl ammonium hydroxide (TMAH), then centrifuged (10 min at 2000G), and filtered before analysis. The dry coffee samples were added 1 mL TMAH and 5 mL deionized water before extraction at 90°C for 3 h before centrifugation and filtration.

The samples were analyzed against a standard addition calibration curve to measure the unknown iodine concentration in the samples. The trueness of the method was verified with the certified reference material and by participation in proficiency tests. All values of certified reference materials were within the accepted area of analysis. The Limit of Quantification (LOQ) was 0.03 μg/100 mL.

### Statistics

The concentrations of iodine in the water samples showed skewed distribution to the right and are thus presented with median and percentiles in addition to crude mean. Statistics were performed in IBM SPSS statistics 27. Values below LOQ were set at 0.015 μg/100 mL with the assumption of normal distribution.

## Results

**Fig. 1:**
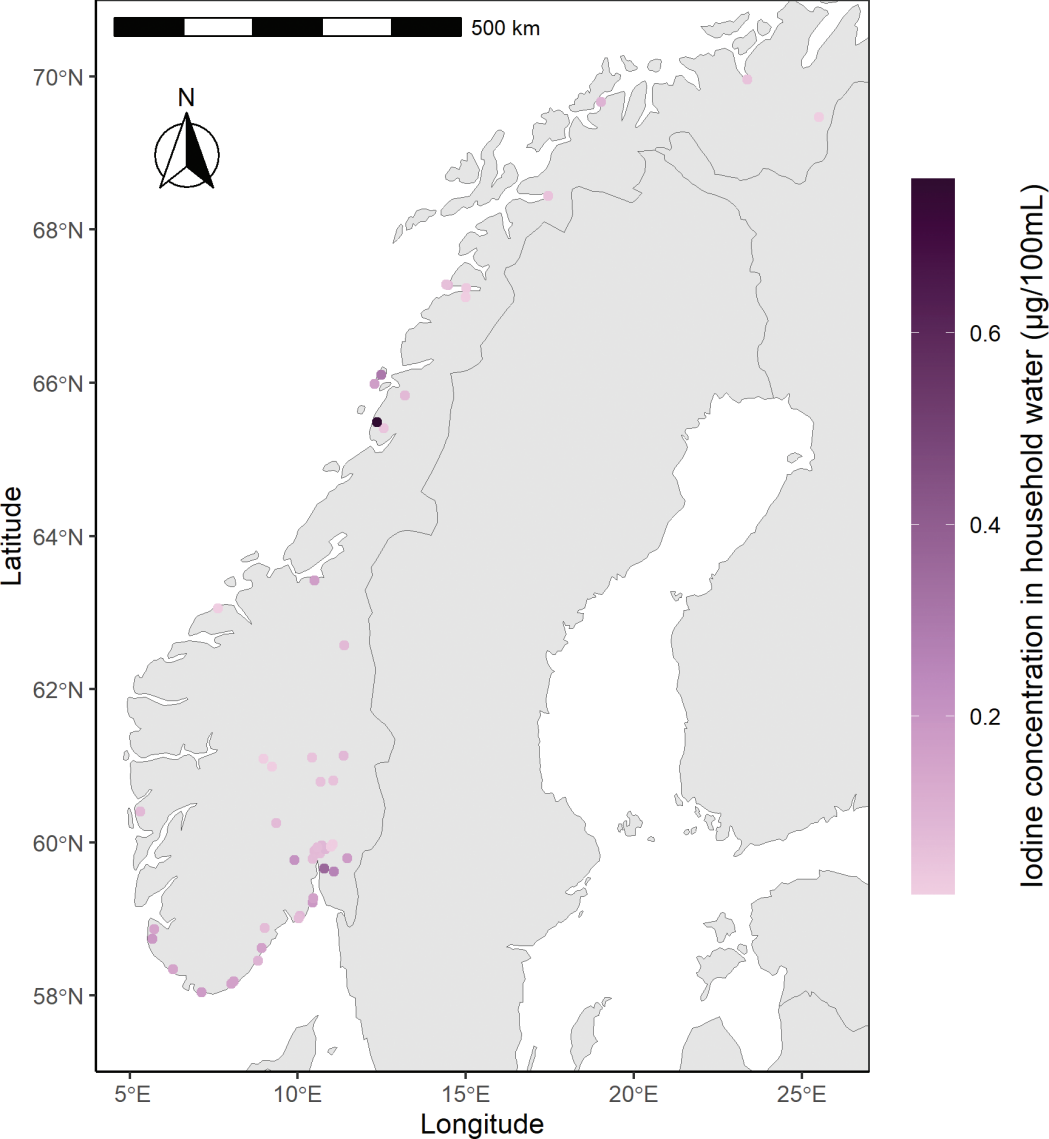
Geographical presentation of the tap water sample locations. Light to dark colors reflect increasing concentrations, ranging from below Limit of Quantification (LOQ) to 0.76 μg/100 mL.

The iodine concentration in drinking water (tap water) ranged from <LOQ to 0.76 μg/100 mL, with median and mean concentrations of 0.08 μg /100 mL and 0.11 μg /100 mL, respectively. Nine samples were below LOQ. The iodine concentration of tap water in different geographical regions in Norway is presented in Table 1 and Fig. 1. When categorizing the locations as coastal (cities or municipalities with sea coastline, *n* = 36) and inland (cities or municipalities without sea coastline, *n* = 20), the median iodine concentration in tap water for coastal regions was significantly higher than samples from inland regions (0.09 vs. 0.06 μg /100 mL, respectively, *P* = 0.02).

**Table 1:** Iodine content in water samples, μg/100 mL

Postal code	Postal area	Region	Iodine, μg/100 mL
0317	Oslo, Oslo	Coastal	0.093
0372	Oslo, Oslo	Coastal	0.086
0667	Oslo, Oslo	Coastal	0.09
1341	Slependen, Viken	Coastal	0.077
1362	Hosle, Viken	Inland	0.069
1389	Heggedal, Viken	Inland	0.071
1433	Ås, Viken	Inland	0.36
1450	Nesoddtangen, Viken	Coastal	0.069
1472	Lørenskog, Viken	Inland	0.015*
1820	Spydeberg, Viken	Inland	0.26
1960	Løken, Viken	Inland	0.18
2007	Kjeller, Viken	Inland	0.015*
2007	Lillestrøm, Viken	Inland	0.015*
2315	Hamar, Innlandet	Inland	0.055
2450	Åmot, Innlandet	Inland	0.083
2608	Lillehammer, Innlandet	Inland	0.052
2815	Gjøvik, Innlandet	Inland	0.057
2900	Fagernes, Innlandet	Inland	0.015*
2900	Fagernes, Innlandet	Inland	0.015*
2966	Vestre Slidre, Innlandet	Inland	0.015*
3113	Tønsberg, Vestfold og Telemark	Coastal	0.16
3133	Færder, Vestfold og Telemark	Coastal	0.19
3260	Larvik, Vestfold og Telemark	Coastal	0.084
3260	Larvik, Vestfold og Telemark	Coastal	0.074
3292	Stavern, Vestfold og Telemark	Coastal	0.072
3300	Hokksund, Viken	Inland	0.21
3359	Eggedal, Viken	Inland	0.074
4313	Sandnes, Rogaland	Coastal	0.15
4340	Bryne, Rogaland	Coastal	0.19
4380	Haugane i Dalane, Rogaland	Coastal	0.15
4521	Lindesnes, Agder	Coastal	0.18
4631	Kristiansand, Agder	Coastal	0.16
4635	Kristiansand, Agder	Coastal	0.16
4818	Færvik, Agder	Coastal	0.099
4900	Tvedestrand, Agder	Coastal	0.16
4980	Gjerstad, Agder	Inland	0.073
5109	Hylkje, Bergen	Coastal	0.08
5817	Bergen	Coastal	0.08
6530	Averøy, Møre og Romsdal	Coastal	0.015*
7059	Jakobsli, Trøndelag	Coastal	0.17
7374	Røros, Trøndelag	Inland	0.078
8010	Bodø, Nordland	Coastal	0.057
8071	Bodø, Nordland	Coastal	0.08
8100	Misvær, Nordland	Coastal	0.015*
8102	Skjerstad, Nordland	Coastal	0.032
8515	Narvik, Nordland	Coastal	0.05
8656	Mosjøen, Nordland	Coastal	0.079
8820	Dønna, Nordland	Coastal	0.3
8850	Herøy, Nordland	Coastal	0.21
8850	Herøy, Nordland	Coastal	0.17
8908	Brønnøysund, Nordland	Coastal	0.76
8960	Velfjord, Nordland	Coastal	0.08
8960	Velfjord, Nordland	Coastal	0.044
9024	Tomasjord, Troms og Finnmark	Coastal	0.099
9516	Alta, Troms og Finnmark	Coastal	0.051
9730	Karasjok, Troms og Finmark	Inland	0.015*
All samples, *n* = 56, median (p25 to p75)			0.08 (0.05 to 0.16)
Coastal regions, *n* = 36, median (p25 to p75)			0.09 (0.07 to 0.16)
Inland regions, *n* = 20, median (p25 to p75)			0.06 (0.02 to 0.08)

Note: Nine samples marked with (*) were below LOQ of 0.03 μg/100 mL and set to 0.015 μg/100 mL based on the assumption of normal distribution. Coastal regions defined as cities and municipalities with coastline.

The iodine concentrations in different commercial mineral waters ranged from <LOQ to 40 μg/100 mL (Table 2).

**Table 2:** Concentration of iodine in commercial mineral water, μg/100 mL

Brand name of mineral water	Iodine, μg/100 mL
Olden, *n* = 1	<LOQ
Farris, *n* = 5, mean (min, max)	38 (33, 40)
Imsdal naturell, *n* = 1	0.13
Smart water, *n* = 1	0.06
Isklar naturell, *n* = 1	0.06

The iodine concentration of brewed filter, instant, and capsule coffees and of coffee brews from coffee shops is presented in Table 3 and ranged from <LOQ to 0.16 μg/100 mL for coffee brews without milk or other additives. For coffee brews with added milk or plant-based beverages, the iodine concentration ranged from <0.8 μg /100 mL to 19 μg /100 mL.

**Table 3:** Concentrations of iodine in brewed coffee, μg/100 mL

Coffee product	Iodine, μg/100 mL		
	Mean	SD	Min, Max
Brewed filtered coffee (*n* = 36)	0.086	0.034	0.048, 0.160
Brewed instant coffee (*n* = 6)	0.084	0.025	0.058, 0.120
Brewed capsule coffee (*n* = 36)	0.042	0.047	0.015, 0.130
Brewed coffee with plant-based milk alternatives (*n* = 8)	9.1	8.5	<0.8, 19.0
Brewed coffee with lactose-free dairy milk (*n* = 1)	17	-	-
Filter ground coffee beans (dry) (*n* = 5)	<LOQ		<LOQ, 0.0036
Instant coffee powder (dry) (*n* = 1)	<LOQ	-	-
Espresso ground coffee beans (dry) (*n* = 2)	<LOQ	-	-

## Discussion

Ensuring adequate iodine intake is important as both intakes below and above the recommended interval are associated with an increase in the risk of disease in the population. Our results showed low iodine concentrations in tap water and coffee samples, and most of the mineral waters, except for one brand of mineral water with high iodine concentration.

The concentrations of iodine in tap water samples in the present study were in line with earlier analyses from Norway in 2004, which found average iodine concentration to be 0.17 μg /100 mL (2). In 1972, Hetland and Simensen sampled and analyzed tap and well waters from 12 different locations along the coast and inland areas of Norway (8, 9). They concluded that overall, there were not significant differences in iodine in water from the sampled locations; however, one inland area and one coastal area showed especially low and high concentrations, respectively. The later study by Dahl et al. in 2004 (2) analyzed iodine in 15 samples from different locations in Norway. They concluded that the samples taken along the coast showed overall higher concentrations of iodine than samples from inland areas. There were, however, variations and locations along the coast that showed low concentrations of iodine in the tap water samples (2). This is in line with the results from the present study, showing that locations with low iodine tap water concentrations were found both along the coast and in inland areas. Likewise, in the other end of the scale, among the six locations with the highest iodine concentrations in tap water, three were inland areas (Hokksund, Spydeberg, and Ås). When categorizing the locations as coastal or inland, the median iodine concentration in tap water from coastal areas was significantly higher than that of the inland areas. However, as this was a convenience sample, and we observed large variations both within coastal and inland areas, results on significant differences should be interpreted with caution.

Most of the water supply systems in Norway use surface water and not ground water (11). This may explain the low-iodine concentration in tap water. In contrast to this, in Denmark, ground water is used for drinking water, and the results from the study by Rasmussen et al. showed higher iodine concentrations in Danish tap water than in the Norwegian tap water samples in the present study (12). In Denmark, the highest iodine concentration was found in tap water samples from eastern Denmark and the lowest in samples from western Denmark with a mean iodine concentration of 1.87 and 0.57 μg /100 mL, respectively. In Sweden, the mean iodine concentration in the surface water was 0.36 μg/100 mL, and 0.5 μg/100 mL in ground water according to the study by Manousou et al. (13).

As for the mineral water, the iodine concentrations in different commercial samples were in the range of tap water, except for one brand, Farris. This mineral water originates from an underground well located beneath a mineral-rich moraine ridge area in the Larvik district of Norway. The iodine concentration was higher than in cow milk, which is an important dietary source of iodine in the Norwegian diet. The intake of a typical bottle of 0.5 L of mineral water will contribute 190 μg of iodine, which is above the recommended daily iodine intake of 150 μg for adults; however, it is below the upper intake level of 600 μg /day for adults (14). The tap water samples from the Larvik area did not show high iodine concentrations and did not differ significantly from tap water samples in other areas of Norway.

Iodine concentrations in black coffee samples reflected the iodine concentration of the water used to brew the beverage and were, in general, low. In comparison, brewed coffee in the Swedish and Danish food composition databases has 3 and 0 μg /100 mL iodine, respectively (15, 16).

We also analyzed the dry coffee (ground beans before brewing), and our results showed that iodine concentration is below the LOQ. This is less than the iodine concentration of ground coffee beans given in the Danish food composition database (0.5 μg /100 g).

In coffee brews with added ingredients, such as milk and plant-based beverages, the iodine concentration was higher. In coffee-added milk, the iodine concentration resembled the concentration found in milk, and the same was observed for coffee-added plant-based beverages. However, the iodine concentration in coffee with plant-based beverages added varied more due to the varying concentration of iodine in the added beverages. In 2020, Dahl et al. analyzed iodine in plant-based beverages and found the concentration to vary from <LOQ to 24 μg /100 mL (17), much depending on the fortification of the product. New plant-based beverage is an expanding commercial food group, and the fortification of these products varies with regard to not only iodine but also other nutrients.

## Conclusions

Iodine concentrations of tap water, black coffee, and most mineral waters were generally low and will therefore most likely not contribute considerably to the iodine intake in population in Norway. However, one brand of mineral water had high concentration of iodine and may contribute significantly to the total dietary iodine intake.
